# 3D printed Gel/PTH@PAHA scaffolds with both enhanced osteogenesis and mechanical properties for repair of large bone defects

**DOI:** 10.1093/rb/rbaf029

**Published:** 2025-05-05

**Authors:** Zhimou Zeng, Ping Song, Xingyu Gui, Bicheng Ake, Taoyu Liu, Hao Liu, Linnan Wang, Lei Wang, Yueming Song, Bo Qu, Changchun Zhou

**Affiliations:** School of Clinical Medicine, Chengdu Medical College, Chengdu 610500, China; Department of Orthopedic Surgery, The First Affiliated Hospital of Chengdu Medical College, Chengdu 610500, China; Department of Orthopedics, Orthopedic Research Institute, West China Hospital, Sichuan University, Chengdu 610041, China; National Engineering Research Center for Biomaterials, College of Biomedical Engineering, Sichuan University, Chengdu 610064, China; School of Clinical Medicine, Chengdu Medical College, Chengdu 610500, China; Department of Orthopedic Surgery, The First Affiliated Hospital of Chengdu Medical College, Chengdu 610500, China; National Engineering Research Center for Biomaterials, College of Biomedical Engineering, Sichuan University, Chengdu 610064, China; School of Clinical Medicine, Chengdu Medical College, Chengdu 610500, China; Department of Orthopedic Surgery, The First Affiliated Hospital of Chengdu Medical College, Chengdu 610500, China; Department of Orthopedics, Orthopedic Research Institute, West China Hospital, Sichuan University, Chengdu 610041, China; Department of Orthopedics, Orthopedic Research Institute, West China Hospital, Sichuan University, Chengdu 610041, China; Department of Orthopedics, Orthopedic Research Institute, West China Hospital, Sichuan University, Chengdu 610041, China; School of Clinical Medicine, Chengdu Medical College, Chengdu 610500, China; Department of Orthopedic Surgery, The First Affiliated Hospital of Chengdu Medical College, Chengdu 610500, China; National Engineering Research Center for Biomaterials, College of Biomedical Engineering, Sichuan University, Chengdu 610064, China

**Keywords:** 3D printing, PA66, scaffolds, large bone defects, osteogenesis

## Abstract

The repair of large bone defects continues to pose a significant challenge in clinical orthopedics. Successful repairs require not only adequate mechanical strength but also exceptional osteogenic activity for successful clinical translation. Composite materials based on polyamide 66 (PA66) and hydroxyapatite have been widely used in various clinical settings. However, existing PA66/hydroxyapatite composites often lack sufficient osteogenic stimulation despite their favorable mechanical properties, which limit their overall clinical efficacy. In this study, we fabricated a polyamide 66/nano-hydroxyapatite (PAHA) scaffold using an extruder and fused deposition modeling-based 3D printing technology. Subsequently, gelatin methacrylamide (GelMA) containing teriparatide (PTH) was incorporated into the PAHA scaffold to construct the Gel/PTH@PAHA scaffold. Material characterization results indicated that the compressive modulus of elasticity and compressive strength of the Gel/PTH@PAHA scaffold were 172.47 ± 5.48 MPa and 25.55 ± 2.19 MPa, respectively. *In vitro* evaluations demonstrated that the Gel/PTH@PAHA scaffold significantly enhanced osteoblast adhesion and proliferation while promoting osteogenic differentiation of BMSCs. *In vivo* studies further revealed that this scaffold notably promoted new bone regeneration in rabbit femoral defects. These findings suggest that the 3D-printed Gel/PTH@PAHA scaffold exhibits excellent mechanical properties alongside remarkable osteogenic activity, thereby meeting the dual requirements for load-bearing applications and bone regeneration. This innovative approach may be a promising candidate for customized orthopedic implants with substantial potential for clinical application.

## Graphical abstract

**Figure:**
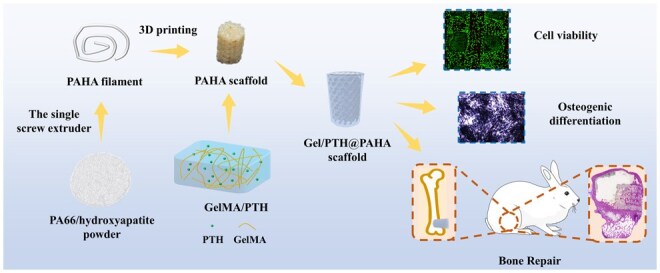

## Introduction

Bone defects resulting from tumors, trauma, diseases and surgical procedures remain a significant challenge in clinical practice [1–3]. In particular, defects that exceed critical size often fail to heal spontaneously, leading to complications such as delayed bone healing and nonunion [4, 5]. Currently, the primary clinical approach for treating bone defects is autogenous or allogeneic bone grafting, though this method has notable drawbacks, including donor site limitations, secondary injuries and high complication rates [6]. Metal implants, such as titanium alloys, offer exceptional mechanical properties, providing robust support at the defect site [7, 8]. However, they are frequently associated with issues such as stress shielding and aseptic loosening. Polyetheretherketone (PEEK) [9], which has an elastic modulus similar to that of human bone, suffers from bioinertness, limiting its ability to integrate effectively with bone tissue [9, 10]. Although the incorporation of bioactive agents, such as calcium phosphate-based compounds [11], can improve the biological functionality of these bioinert materials, such modifications have not yet seen widespread clinical application and may increase manufacturing costs and associated risks [12, 13].

Polyamide66/nano-hydroxyapatite (PAHA), a biomimetic organic/inorganic composite material, has been extensively validated in the clinic [14–16]. The excellent mechanical properties of PA66 closely resemble those of natural bone, and its non-degradability avoids the issues associated with the degradation and acidic by-products of biodegradable materials like polylactic acid (PLA) and polycaprolactone (PCL). This characteristic reduces risks and provides long-term stability [17, 18]. In addition, PA66 circumvents the stress shielding issue inherent in metallic materials like titanium alloy while overcoming the low toughness and brittleness characteristic of ceramic materials. Nano-hydroxyapatite (n-HA) enhances the bioactivity of PA66 by offering good bone conductivity and potential osteoinductivity [19, 20]. However, the dense PA66/n-HA composite lacks a porous structure, which limits cell growth, vascularization and new bone formation [21, 22]. Traditional fabrication methods have difficulty producing scaffolds with customized 3D structures and interconnected pores [23]. Recently, 3D printing technology has garnered significant attention in the field of bone repair, owing to its ability to create intricate, tailored structures [24–27]. In particular, PA66/n-HA scaffolds fabricated using extrusion-based 3D printing have been widely studied for bone repair applications [28, 29]. However, for critical-size bone defects, where extensive damage to bone tissue and blood vessels occurs, new bone growth is typically confined to the interface between the implant and the defect. As a result, large-scale new bone formation within the implant itself may not be induced, often leading to suboptimal repair outcomes.

Teriparatide (PTH) is an active recombinant 1–34 amino acid fragment of human parathyroid hormone PTH, which has been approved by the US Food and Drug Administration to promote bone synthesis for the treatment of bone loss [30, 31]. Low-dose PTH released intermittently activates the cAMP/PKA, Wnt/β-catenin and MAPK signaling pathways, promoting the expression of osteogenic genes such as alkaline phosphatase (ALP) and osteopontin (OPN), thereby enhancing osteogenesis. In contrast, sustained high-dose PTH release activates the RANKL/NF-κB pathway, upregulates osteoclast-related genes like Bcl-2 and TRAP and enhances osteoclastogenesis [32, 33]. At present, PTH is mainly administered subcutaneously in clinical practice, but the cost of use is high, and high-dose use has potential systemic side effects. Gelatin methacrylamide (GelMA) is gelatin based hydrogel, which has adjustable physical and chemical properties, good biodegradability and biocompatibility [34]. Its porous network structure provides sufficient load space for drugs, enabling drugs to be evenly dispersed in the hydrogel, enabling controlled drug release [35]. Therefore, PTH loaded with GelMA hydrogel can be administered on the bone defect *in situ*, which can greatly reduce the use of PTH and has the advantages of low cost and small side effects. A recently developed methodology for scaffold fabrication, often termed the ‘host–guest’ system, has gained traction in tissue engineering applications [36–38]. This system provides mechanical support through the polymer host component, while introducing functionalized guest components to induce osteogenic effects. Therefore, constructing a scaffold with PAHA as the host component and Gel/PTH as the functionalized guest has the potential to solve the challenge of large bone defects.

The critical challenge in repairing large bone defects lies in balancing mechanical strength and osteogenic activity. Existing PA66/hydroxyapatite composites lack sufficient osteogenic stimulation despite good mechanical properties, which limits their clinical efficacy. In this study, Gel/PTH@PAHA porous scaffold with enhanced osteogenic performance and high mechanical properties was successfully fabricated via extrusion-based fused deposition modeling (FDM) 3D printing and incorporated GelMA hydrogel loaded with PTH. The physicochemical properties of the Gel/PTH@PAHA scaffolds were characterized, and their biocompatibility and osteogenic inductive potential were evaluated. Finally, a critical femoral defect model was established in rabbits, and the bone regeneration capacity of the scaffold was evaluated *in vivo*. This scaffold may be a promising candidate for customized orthopedic implants with significant potential for clinical application.

## Materials and methods

### Preparation and characterization of PAHA filament

**Figure 1.**
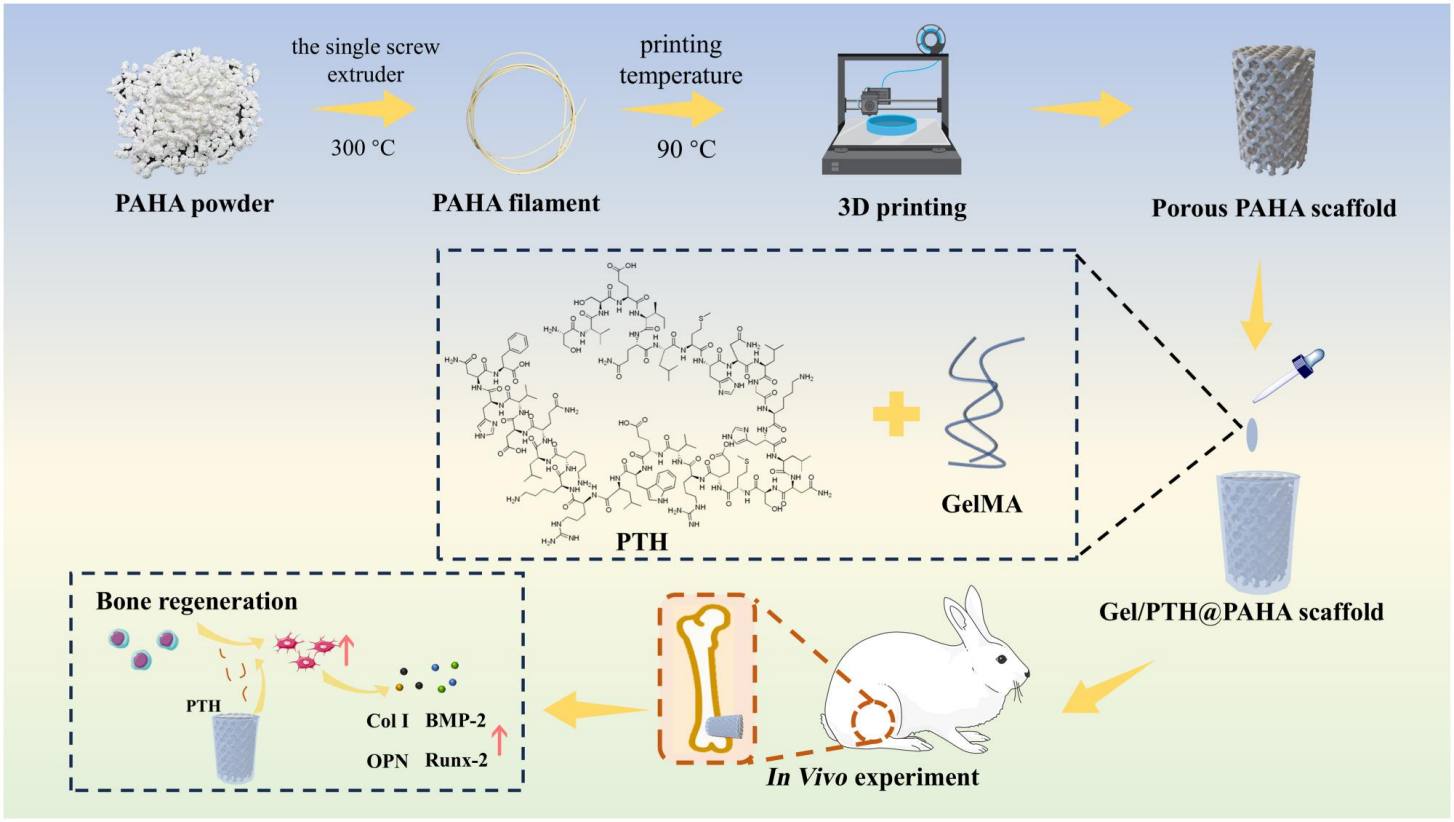
Schematic illustration of the preparation and bone repair application of the Gel/PTH@PAHA scaffold.

As shown in Figure 1, porous PAHA scaffolds were fabricated using FDM-based 3D printing technology in this study, with PTH encapsulated in GelMA. The Gel/PTH solution was then injected into the porous PAHA scaffolds to form the Gel/PTH@PAHA scaffolds. PAHA powder composite with a weight ratio of 3:7 was purchased from Sichuan Guona Technology Co., Ltd [17]. Firstly, the powder PAHA was dried in a vacuum drying oven at 80°C for 24 h. The chamber of the single-screw extruder was then preheated to a temperature range of 260–300°C, and the nozzle temperature was set to 300°C. Finally, the composite powder was added to a single screw extruder and heated to melt before being extruded into 3D printing filament. Phase analysis of PAHA powder was conducted by X-ray diffraction (XRD, Philips X'Pert 1, Netherlands). The surface morphology of both the PAHA powder and PAHA filament was observed using scanning electron microscopy (SEM, JSM-5900LV, JEOL).

### Preparation and characterization of GelMA

The detailed synthesis method has been previously described [19]. Briefly, type A porcine skin gelatin (VWR, USA) was dissolved in a sodium carbonate–bicarbonate buffer at 60°C. Methacrylic anhydride (MA, Huaxia, China) was then added to the gelatin solution at a MA/gelatin feed ratio of 0.1/1 and reacted for 3 h at 50°C. Subsequently, the solution was dialyzed using a dialysis membrane with distilled water for 3 days. Finally, the GelMA solution was freeze-dried to obtain sponge GelMA. The synthesized GelMA samples were dissolved in D_2_O and analyzed using 400-MHz nuclear magnetic resonance (NMR) spectroscopy (Bruker AVANCE AVII-400 MHz, Switzerland).

### 3D printing of PAHA scaffolds

A disc-shaped porous scaffold with a diameter of 10 mm and a height of 2 mm was designed using Solidworks software and an STL file of the 3D model was exported. Subsequently, the 3D printing slicing software Creatware was utilized to establish the printing path for the porous scaffold. The printing parameters were set as follows: a layer height of 0.2 mm, a printing speed of 40 mm/s, a filling degree of 50%, an extrusion temperature of 290°C and a hot bed temperature of 100°C. Finally, the Gcode file was exported from the software and imported into the CreaBot PEEK 300 3D printer for the printing of the porous scaffolds.

### Preparation of Gel@PAHA and Gel/PTH@PAHA scaffolds

Firstly, teriparatide (Shifeng Biotechnology, China) was dissolved in ultrapure water at a concentration of 100 μg/ml [39]. Subsequently, GelMA with a concentration of 6 w/v% was prepared from this solution and designated as Gel/PTH. Then, the 6 w/v% GelMA solution and the Gel/PTH solution were separately injected into PAHA scaffolds, followed by UV irradiation of the scaffolds to prepare Gel@PAHA scaffolds and Gel/PTH@PAHA scaffolds, respectively. Finally, the scaffolds were frozen in a refrigerator at −20°C and subsequently lyophilized to obtain the freeze-dried scaffolds (Gel@PAHA and Gel/PTH@PAHA).

### Characterization of 3D printed PAHA scaffolds

The 3D printed scaffolds were freeze-dried, followed by gold spraying treatment. The surface morphology of the 3D printed scaffold was observed using a scanning electron microscope (SEM, JSE-5900LV, Japan). Meanwhile, the surface elements of the scaffolds were investigated with energy-dispersive X-ray spectrometry (EDS). Differential scanning calorimeter (DSC, Q2000, TA Instruments, USA) was used to detect the thermal properties of samples. The compression performance of the 3D printed scaffolds was tested using a universal mechanical testing machine at a compression speed of 1 mm/min.

### *In vitro* experiment

#### Cell culture and scaffold preparation

Under simulated *in vivo* conditions (37°C, 5% CO_2_ incubator), mouse embryonic osteoblast precursor cells (MC3T3-E1) in quantities of one million were cultured in α-MEM medium (Gibco, USA) supplemented with 10% fetal bovine serum (Gibco, USA) and 1% bispecific antibiotic solution (penicillin 100 U/ml, streptomycin 100 μg/ml, Hyclone, USA). To facilitate cell experiments, all composite scaffolds were designed with a diameter of 10 mm and a height of 2 mm. At least three parallel samples were prepared for each scaffold to ensure experimental reproducibility. These scaffolds underwent sterilization via ethylene oxide treatment.

#### Cell viability assessment

Cells were seeded onto all scaffold surfaces at a number of 5 × 10^4^ cells and were then placed in a 24-well plate. Fresh culture medium was replaced every 48 h to sustain optimal cell growth conditions. On the 1st, 3rd and 7th days of cultivation, cell viability was assessed using fluorescein diacetate (FDA, Sigma, USA) and propidium iodide (PI, Sigma, USA). Live cells were stained green, while dead cells were stained red. These staining results were observed under a laser confocal microscope (Carl Zeiss, Germany). Additionally, the CCK-8 assay kit (Beyotime, China) was employed to measure cell proliferation at these same time points, with the number of cells being quantified by measuring the OD value at a wavelength of 450 nm.

#### Cell proliferation and morphological observation

To further evaluate the proliferation and adhesion morphology of cells on the scaffold, the cytoskeleton and nucleus were stained with 4′,6-diamidino-2-phenylindole (DAPI, Sigma, USA) and phalloidin (Sigma, USA), followed by observation under a laser confocal microscope.

#### In vitro osteogenic differentiation

To investigate the effect of scaffolds on osteogenic differentiation in cells, 5 × 10^5^ rabbit bone marrow mesenchymal stem cells (BMSCs) were cocultured with scaffolds for evaluation. The cells were initially pre-cultured in α-MEM complete medium for 3 days, which was then replaced with osteogenic induction medium consisting of high-glucose DMEM medium (Gibco, USA) supplemented with 10% FBS, 1% PS, 0.008 μg/ml β-glycerophosphate, 1.76 μg/ml ascorbic acid and 0.66 μg/ml dexamethasone. After 14 days of osteogenic induction culture, osteogenic differentiation and matrix mineralization ability were assessed through alkaline phosphatase staining (ALP, Beyotime, China) and alizarin red (ARS, Beyotime, China) staining. The samples were then washed with PBS (Gibco, USA), and the staining was observed under a stereomicroscope. To validate the osteogenic induction ability of the scaffold at the molecular level, the mRNA transcription levels of osteogenic-related genes (Col I, OPN, BMP-2 and Runx-2) were detected using quantitative real-time polymerase chain reaction (qRT-PCR). After 14 days of cultivation in osteogenic induction medium, cells were collected and RNA was extracted using a reagent kit. Subsequently, a cDNA kit (Bio-Rad, USA) was used to reverse transcribe the RNA into cDNA. Finally, SYBR Green real-time PCR was employed to detect the osteogenic target genes.

### *In vivo* experiments

The *in vivo* experiment was approved by the Ethics Committee of Experimental Animals at West China Hospital of Sichuan University (Approval No. KS2021544). The New Zealand white rabbits (2.2–2.5 kg) used in this experiment were purchased from Chengdu Dashuo Experimental Animal Co., Ltd. Surgical instruments were sterilized using a high-temperature and high-pressure sterilization pot. Firstly, pentobarbital sodium with a concentration of 20 mg/ml was injected into the rabbit ear vein at a dose of 2 ml/kg. After the rabbit was fully anesthetized, cylindrical defects with a diameter of 6 mm and a height of 9 mm were made on both femoral condyles, and then scaffolds were implanted into the defect sites. The wound was carefully sutured, and gentamicin sulfate was injected into the rabbit thigh to prevent infection. After 1 and 2 months, rabbits were sacrificed by injecting excessive pentobarbital sodium. Subsequently, samples were taken together with the femur and fixed with 4% paraformaldehyde.

### Micro-CT detection and analysis

The sample, which had been soaked in 4% paraformaldehyde, was taken out and cleaned with PBS, fixed in the sample tube and then subjected to a Micro-CT scan (VivaCT 80, SCANCO Medical AG, Switzerland). The Dicom files obtained from the scan were processed using Mimics 19.0 software, and a model of the intermediate bone defect area was created based on the actual bone tissue threshold. The analysis indicators included the new bone volume fraction (BV/TV), trabecular thickness (Tb.Th), trabecular separation (Tb.Sp) and trabecular number (Tb.N).

### Organizational analysis

The sample soaked in 4% paraformaldehyde was taken out and cleaned with PBS. Subsequently, the sample underwent gradient dehydration in 30%, 50%, 70%, 85%, 95% and 100% alcohol for 1 h each. It was then embedded and cured using a photocuring resin and sliced with a hard tissue slicer (Leica, SM2500E, Germany). The slices were ground down to a thickness of 15 μm using a membrane machine and finally stained with hematoxylin and eosin (H&E) for histological evaluation.

### Statistical analysis

All data were presented as mean ± standard deviation (SD). Statistical analysis was conducted using one-way ANOVA using GraphPad Prism software (GraphPad Software Inc, USA). The statistical significance levels were set as *p*< 0.05 (*), *p* < 0.01 (**), *p* < 0.001 (***) and *p* < 0.0001 (****).

## Results and discussion

### Preparation and characterization of PAHA filament and GelMA hydrogel

**Figure 2.**
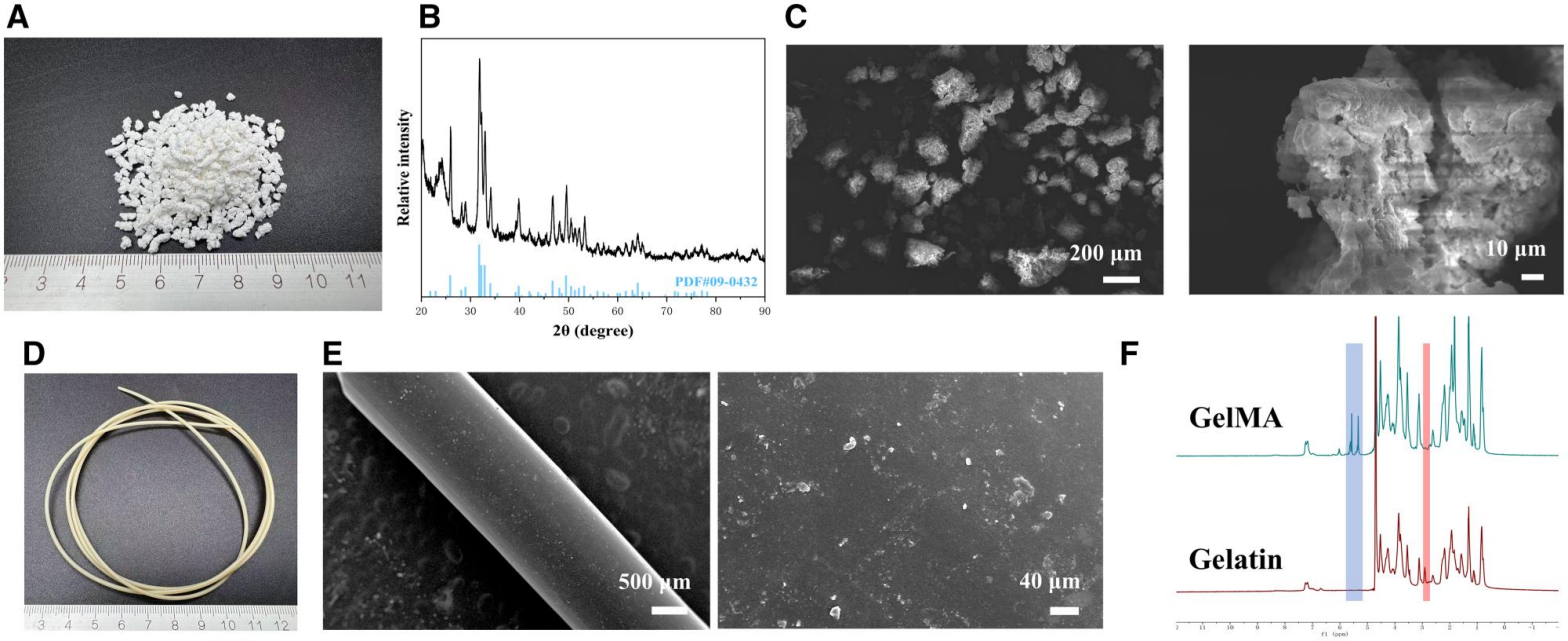
Characterization of PAHA powder/filament and GelMA. (**A**) PAHA powder. (**B**) XRD diffraction patterns of PAHA powder. (**C**) SEM images of PAHA powder. (**D**) PAHA filament. (**E**) SEM images of PAHA filament. (**F**) ^1^H NMR spectra of gelatin and GelMA.

PA66/n-HA (PAHA) has been extensively used as an orthopedic implant material in clinical treatments due to its cortical bone-like mechanical properties and exceptional osteogenic activity. However, progress in the use of 3D-printed, custom-made PAHA scaffolds for bone repair and their clinical applications have been limited. In this study, based on our previous research, the PAHA powder was fed into an extruder to prepare 3D printing PAHA filaments, as shown in Figure 2A. XRD analysis (Figure 2B) revealed that the characteristic peaks of the PAHA powder closely matched those of hydroxyapatite, confirming a substantial presence of hydroxyapatite in the PAHA powder. SEM images of the PAHA powder surface also showed uniformly dispersed nanoscale hydroxyapatite (Figure 2C). After being extruded through a nozzle heated to 300°C, the PAHA powder was thermally formed into filaments with a diameter of 1.75 ± 0.5 mm. The surface of the PAHA filaments was smooth, with no visible cracks or bubbles. Additionally, as shown in Figure 2F, GelMA, a hydrogel that serves as a carrier for PTH, was successfully synthesized. Comparison with the ^1^H NMR spectrum of gelatin demonstrated that, after adding methacrylic anhydride (MA), a characteristic resonance peak for acrylic protons (2H) (5.2–5.7 ppm) from the methacrylamide grafts in GelMA appeared, while the characteristic signals for the methylene protons (2.8–2.95 ppm) of lysine were significantly reduced. After blending GelMA solution with PTH, it can be directly cured into Gel/PTH hydrogel using ultraviolet light. The high-water content of this hydrogel provides a microenvironment similar to that within a biological body, allowing for the storage and release of the drug under conditions that mimic those of the body [40].

### Preparation and characterization of 3D printed Gel/PTH@PAHA scaffolds

**Figure 3.**
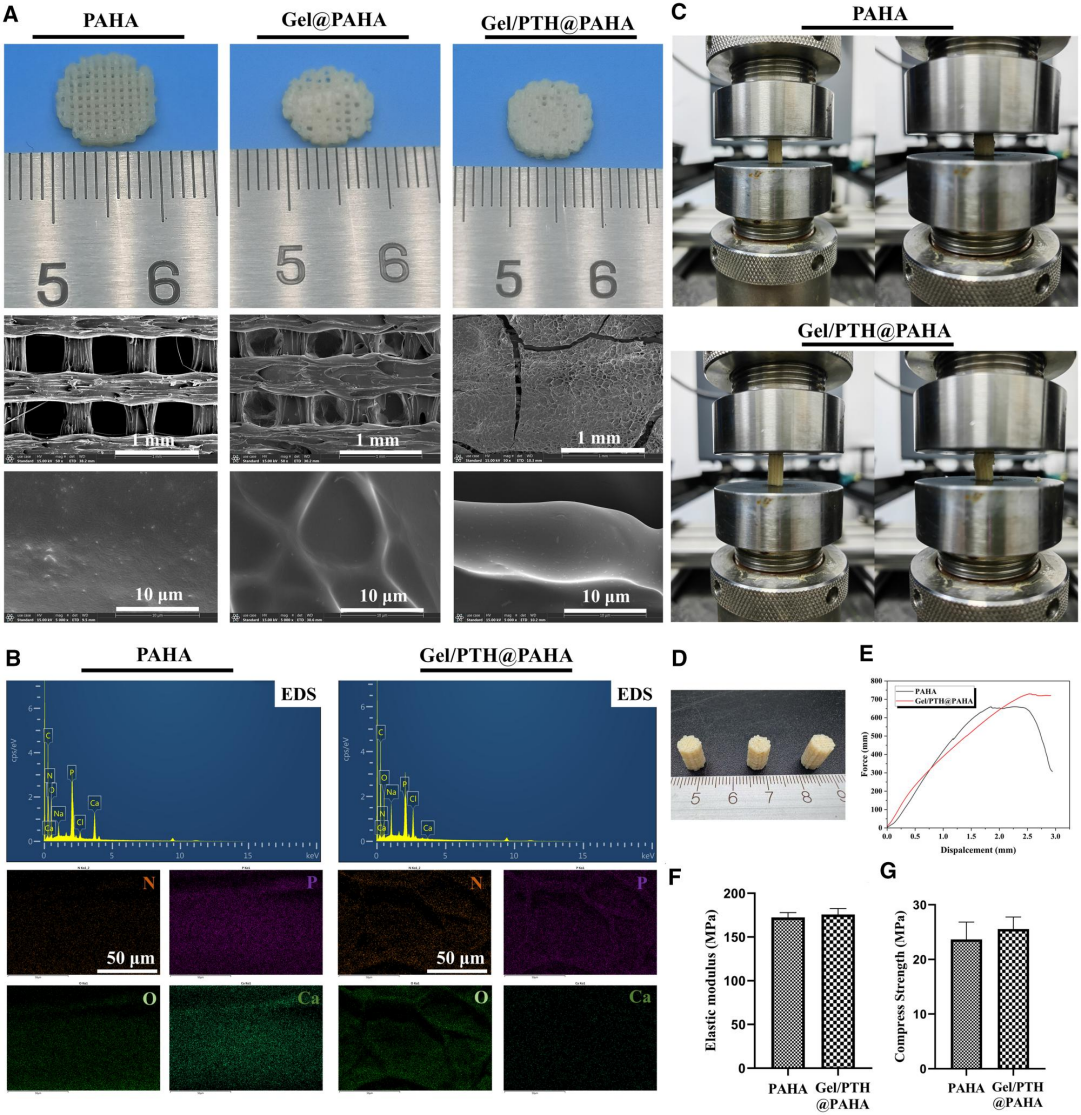
Characterization of PAHA, Gel@PAHA and Gel/PTH@PAHA scaffolds. (**A**) 3D printed scaffolds and their SEM images. (**B**) EDS energy spectrum on the surface of the scaffold, EDS elemental mapping images of the cross-sections of scaffold. (**C**) Mechanical test of 3D printed scaffolds. (**D**) 3D printed scaffolds. (**E**) Force and displacement curves of 3D printed scaffolds. (**F**) Elastic modulus of 3D printed scaffolds. (**G**) Compress strength of 3D printed scaffolds.

After PAHA filaments were prepared via melt extrusion, porous PAHA scaffolds were fabricated using 3D printing technology based on fused deposition modeling. Figure 3A illustrates the shapes and morphologies of the 3D-printed PAHA, Gel@PAHA and Gel/PTH@PAHA scaffolds. All scaffolds exhibited well-distributed and uniform porous structures, as the 3D printing process enabled the modulation of porosity and pore size by adjusting the fill rate and pore architecture. Within the PAHA scaffolds, nano-hydroxyapatite was uniformly dispersed within the PA66 polymer, and some cracks parallel to the printing direction were observed on the scaffold surface, potentially resulting from rapid extrusion and nozzle movement during printing. For the Gel@PAHA scaffolds, the injection of hydrogel slightly decreased the macropore size. Similarly, the pore structure of the Gel/PTH@PAHA scaffolds was filled with Gel/PTH, and compared to the Gel@PAHA scaffolds, the hydrogel surface of these scaffolds was adorned with numerous nanoscale PTH particles, indicating successful PTH loading onto the PAHA scaffolds. Additionally, the porous polymer network formed by the Gel/PTH hydrogel allows for the encapsulation of PTH within its matrix. The release of PTH can occur through diffusion or via degradation of the hydrogel network. This controlled release mechanism is particularly suited for situations where a slow or sustained release of the drug is necessary to maintain therapeutic concentrations. Despite the favorable osteogenic activity of PAHA, in cases of severe bone defects, the bone tissue and blood vessels at the defect site are severely damaged and lost, resulting in a scarcity of stem cell sources and vascular deficiency. Consequently, PAHA may only provide minimal osteogenic activity, yielding suboptimal therapeutic outcomes for the treatment of severe bone defects. The EDS spectral scanning of the scaffold in Figure 3B revealed that PAHA scaffold and Gel/PTH@PAHA scaffold had been evenly distributed with their various elements. However, it was found that Gel/PTH@PAHA scaffold had relatively few calcium and phosphorus elements associated with hydroxyapatite, which may have been the reason why a large amount of Gel/PTH was wrapped around the surface of the scaffold.

The mechanical properties of bone repair scaffolds were found to play a significant role in bone repair. Compression mechanical properties tests were conducted on PAHA scaffolds and Gel/PTH@PAHA scaffolds (Figure 3C and D), revealing that neither type exhibited brittle fracture, but compression resulted in longitudinal densification and reduced porosity. The stress–strain curve results (Figure 3E) indicated that both scaffolds initially exhibited rigid linear response, followed by yielding. The elastic moduli of the PAHA scaffold and Gel/PTH@PAHA scaffold were determined to be 172.47 ± 5.48 MPa and 175.68 ± 6.84 MPa, respectively, with compressive strengths of 23.67 ± 3.18 MPa and 25.55 ± 2.19 MPa, respectively. The Gel/PTH@PAHA scaffold exhibited slightly superior compressive mechanical properties compared to the PAHA scaffold, potentially due to the hydrogel filling enhancing the mechanics. Considering the high elastic modulus of most metal bone implants and the brittle nature of ceramic bone implants, 3D-printed porous scaffolds based on PAHA materials effectively met the mechanical requirements for bone repair. With a compressive strength ranging from 11 to 24 MPa, the 3D-printed Gel/PTH@PAHA scaffold significantly surpassed that of cancellous bone [41, 42], suggesting its potential for application in load-bearing bone repair areas.

### *In vitro* cell experiment

**Figure 4.**
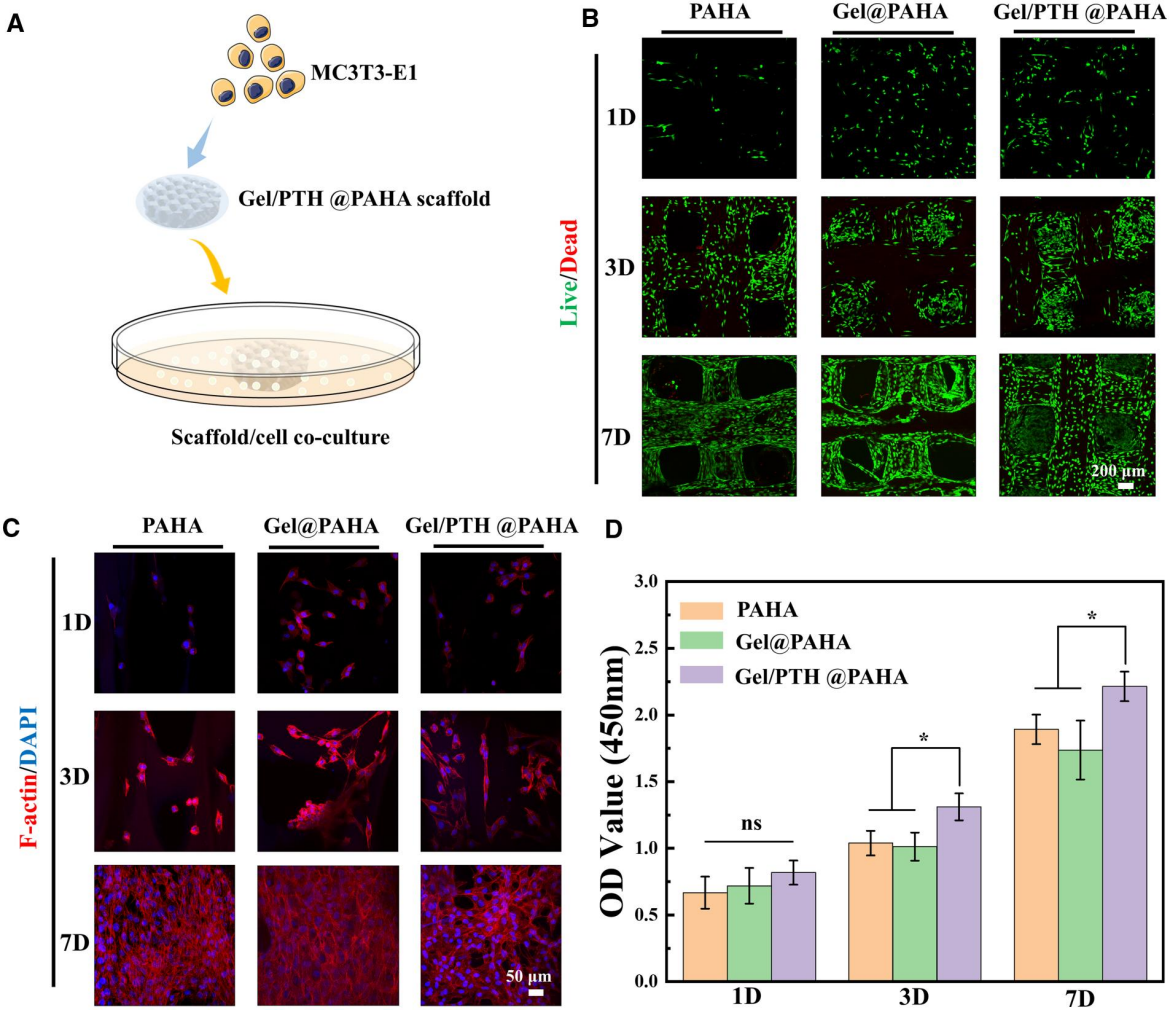
Evaluation of cytocompatibility, adhesion and proliferation of MC3T3-E1 on 3D printed scaffolds. (**A**) Schematic diagram of scaffold cell co-culture for evaluating biocompatibility. (**B**) Confocal microscopic images of live/dead staining of MC3T3-E1 cells on the scaffold. (**C**) Confocal microscopic images of rhodamine phalloidin/DAPI staining of MC3T3-E1 cells on the scaffold. (**D**) Quantification of cell viability and toxicity on scaffolds, *p*<0.05(*).

To evaluate the biocompatibility and cell adhesion of 3D-printed PAHA, Gel@PAHA and Gel/PTH@PAHA scaffolds, *in vitro* co-culture experiments of scaffolds with cells were conducted (Figure 4A). As is shown in Figure 4B, fluorescent images were acquired with live cells stained with FDA and dead cells stained with PI. One day post-inoculation onto the scaffold surface, abundant green fluorescence and sparse red fluorescence were observed across the scaffold, signifying excellent biocompatibility. Over the course of 4 and 7 days of culture, cells proliferated and migrated rapidly on the scaffold with green fluorescence progressively delineated the porous scaffold's outer contour. As shown in Figure 4C, the phalloidin-stained actin (red fluorescence) and DAPI-stained cell nuclei (blue fluorescence) on the scaffolds were clearly visible, indicating that the cells had adhered and spread on the scaffold surface after 1 day of co-culture. By day 4 and day 7 of co-culture, the number of cells significantly increased, forming a distinct phenomenon of cell colony formation. Furthermore, in Figure 4D, quantitative results using the CCK-8 reagent demonstrated that the Gel/PTH@PAHA scaffold promoted cell proliferation and viability more effectively than the other two scaffolds.

**Figure 5.**
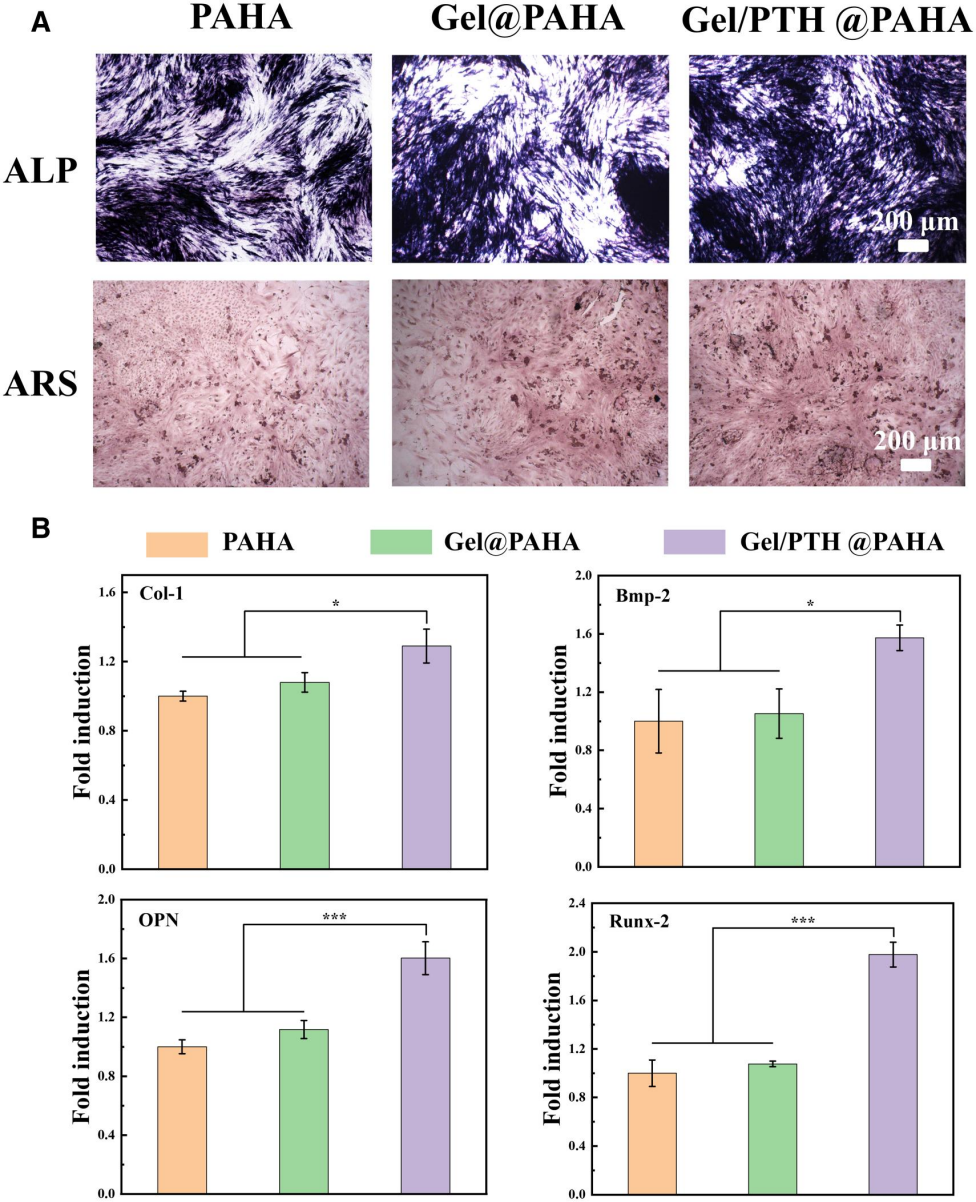
Evaluation of osteogenic differentiation promotion by 3D-printed scaffolds *in vitro*. (**A**) ALP staining and ARS experiment at 14 days. (**B**) Quantitative Col-I, BMP-2, OPN and Runx-2 gene expression of BMSCs by real-time PCR analysis, *p*<0.05(*), *p*<0.001(***).

Subsequently, the scaffold was evaluated for promoting bone differentiation *in vitro*. As shown in Figure 5A, distinct cell spreading patterns detected by ALP staining and calcium nodules visualized by ARS staining can be observed in all three groups, but the positive expression area of the Gel/PTH@PAHA scaffold was greater than that of the other scaffolds, indicating that more BMSCs were induced to become osteoblasts and secrete osteogenic factors. The PAHA and Gel@PAHA scaffolds effectively promoted osteogenic differentiation of BMSCs due to the release of calcium phosphate ions by hydroxyapatite. In addition, the expression of osteogenic-related genes was detected after co-culturing the scaffold and BMSCs for 14 days. As shown in Figure 5B, Gel/PTH@PAHA scaffolds loaded with PTH had greater ability to promote osteogenic gene expression than other scaffolds. Therefore, compared to PAHA and Gel@PAHA scaffolds, Gel/PTH@PAHA had superior osteogenic induction ability.

### *In vivo* experiments

**Figure 6.**
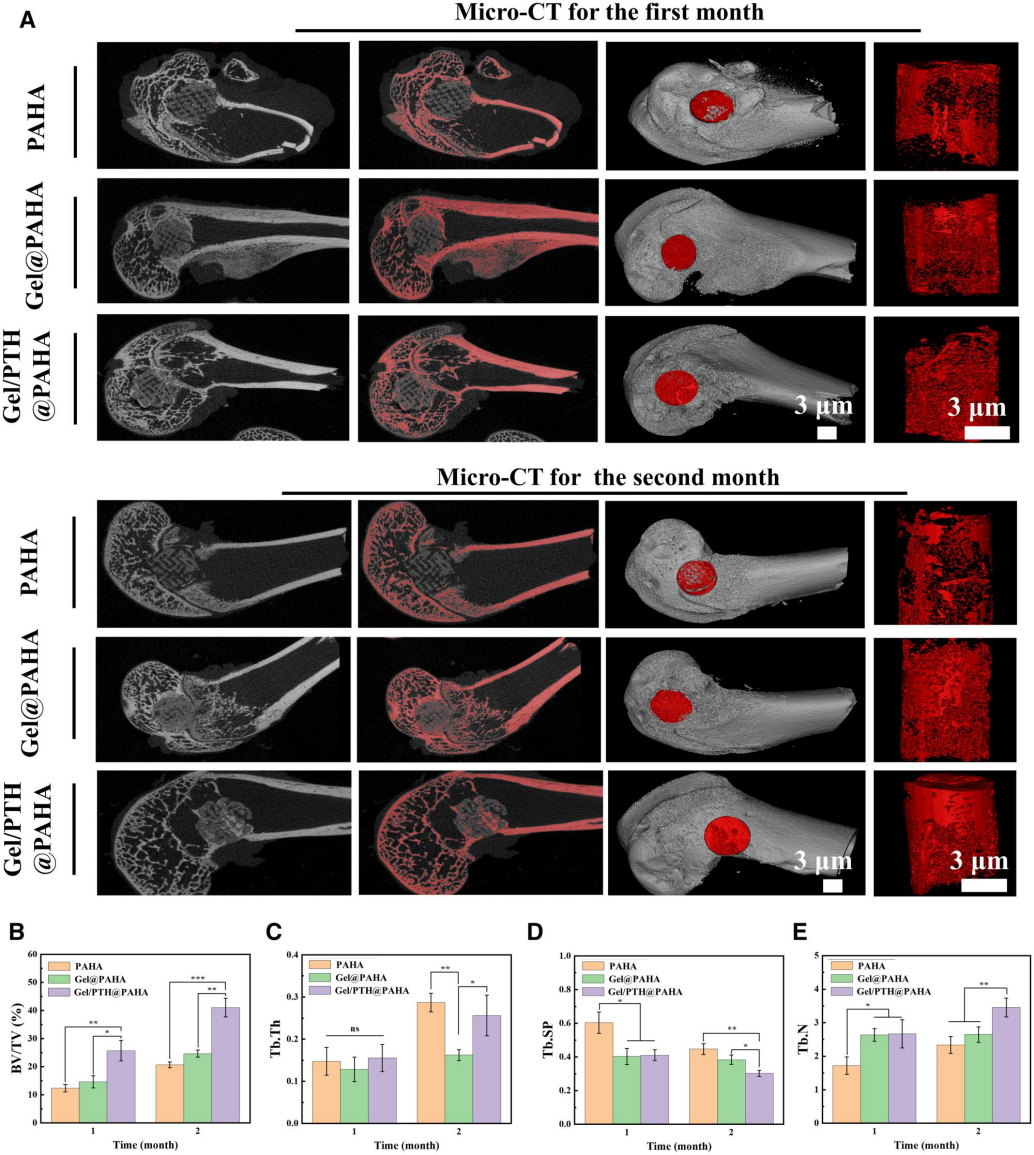
Micro-CT evaluation of bone regeneration *in vivo*. (**A**) Micro-CT reconstructions showing cross-sectional views of femoral defects and Micro-CT reconstructions of newly formed bone. (**B**) Bone volume fraction (BV/TV) of newly formed bone. (**C**) Trabecular thickness (Tb.Th). (**D**) Trabecular separation (Tb.Sp). (**E**) Trabecular number (Tb.N), *p*<0.05(*), *p*<0.01(**), *p*<0.001(***).

To further evaluate the *in vivo* osteogenic performance of the 3D-printed Gel/PTH@PAHA scaffolds, a rabbit femoral bone defect model was employed. Micro-CT scanning and reconstruction of the bone defect sites were conducted at 1 and 2 months postoperatively. In Figure 6A, it was observed that all 3D-printed scaffold groups exhibited good integration with the bone tissue, which was attributed to the excellent osseointegration provided by the hydroxyapatite in the scaffolds. Although new bone tissue ingrowth was observed at the edges of all scaffolds after the first month, the Gel/PTH@PAHA scaffolds exhibited significantly more new bone formation and deeper ingrowth into the interior of the scaffolds. This indicated that the Gel/PTH@PAHA scaffolds loaded with PTH possessed stronger early osteogenic capacity. Furthermore, quantitative analysis of the volume and quality of new bone formation in the bone defect sites was performed based on micro-CT. Compared to the PAHA and Gel@PAHA scaffolds, the Gel/PTH@PAHA scaffolds demonstrated a significant increase in the bone volume fraction (BV/TV) at both 1 and 2 months post-surgery, with a particularly substantial osteogenic amount after the first month. Although there was some degree of new bone regeneration in the PAHA and Gel@PAHA scaffolds, the repair effect was not satisfactory after 2 months. Given the presence of trabecular bone structure in the femoral region, quantitative analysis of the trabecular bone structure was conducted. Trabecular thickness (Tb.Th), representing the average thickness of trabeculae, was found to be related to the pore structure of the porous scaffolds [29]. In this study, as shown in Figure 6C, the average thickness in the Gel/PTH@PAHA scaffolds was higher than that in the Gel@PAHA group, possibly due to the extensive new bone formation at the scaffold edges during the early stages. Trabecular spacing (Tb.Sp), indicating the average width of the marrow cavity between trabeculae, was found to be smaller in the Gel/PTH@PAHA scaffolds compared to other scaffolds in Figure 6D. This may be because the PTH released from the scaffolds promoted new bone regeneration and increased trabecular density. Trabecular number (Tb.N), representing the number of intersections between bone and non-bone tissues, was significantly higher in the Gel/PTH@PAHA scaffoldFis than in other scaffolds in Figure 6D, which was also a result of the osteogenic promotion by the Gel/PTH@PAHA scaffolds.

**Figure 7.**
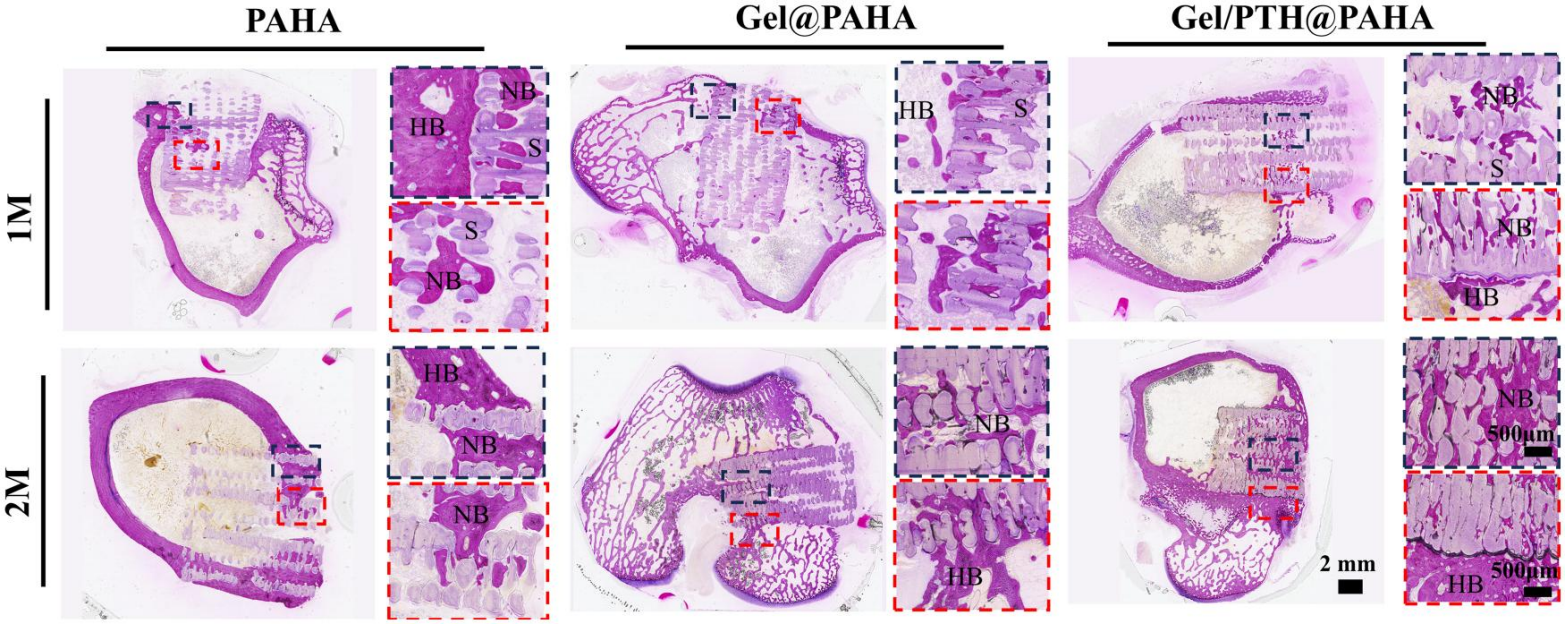
H&E staining of the bone defect at 1 and 2 months after 3D printed scaffolds implantation. HB refers to host bone; NB refers to new bone; S refers to scaffold.

Hard tissue sections of each femoral sample were stained with H&E staining (Figure 7) to further evaluate the *in situ* bone repair capability of the 3D-printed scaffolds. It was observed that the edges of the PAHA scaffolds were tightly integrated with the bone tissue. Two months postoperatively, a small amount of new bone tissue grew into the interior, but the interior showed minimal new bone formation. Compared to the PAHA scaffolds, the Gel@PAHA scaffolds exhibited more regenerated bone two months postoperatively, possibly due to the GelMA hydrogel within the scaffolds providing a favorable adhesion interface and serving as a bridge for cell migration. For the Gel/PTH@PAHA scaffold group, one month postoperatively, new bone tissue had already grown deeply into the core area, filling and sealing the defect. By two months postoperatively, new bone had infiltrated the porous structure of the Gel/PTH@PAHA scaffolds, indicating that PTH loaded within the GelMA hydrogel significantly promoted new bone regeneration. Therefore, the Gel/PTH@PAHA scaffolds exhibited the best performance in the repair of *in situ* bone defects.

## Conclusion

This study developed Gel/PTH@PAHA scaffolds for repairing large weight-bearing bone defects, systematically evaluating their mechanical properties and osteogenic potential. Porous PAHA scaffolds were fabricated via 3D printing, followed by functionalization with GelMA-hydrogel-encapsulated PTH. Mechanical testing revealed that Gel/PTH@PAHA scaffolds exhibited compressive elastic modulus of 175.68 ± 6.84 MPa and compressive strength of 25.55 ± 2.19 MPa. *In vitro* assays demonstrated that Gel/PTH@PAHA scaffolds significantly promoted osteoblast adhesion, proliferation and osteogenic differentiation of BMSCs, underscoring their superior biocompatibility. *In vivo* experiments in rabbit femoral defects showed that Gel/PTH@PAHA scaffolds induced substantial bone regeneration, outperforming PAHA and Gel@PAHA controls. Collectively, these 3D-printed scaffolds integrate robust mechanical performance with enhanced osteogenic activity, presenting substantial potential for customized orthopedic implants in clinical settings.

## Funding

This work was partially supported by the National Natural Science Foundation of China (32471474), Sichuan Science and Technology Program (2024YFFK0063, 2024YFHZ0125), Natural Science Foundation of Sichuan (2024NSFSC1815), Youth Innovation Project of Sichuan Medical Association (Q2024093), Sichuan Province Medical Research Project Plan (S23035), Science and Technology Project of Health Commission of Sichuan Province (23LCYJ032), General Research Project of Sichuan Provincial Administration of Traditional Chinese Medicine (2024MS026), China Postdoctoral Science Foundation (GZC20231817), Post-Doctor Research Project, West China Hospital of Sichuan University (2024HXBH122).

*Conflicts of interest statement.* The authors declare that there is no conflict of interest.

## References

[1] Dalisson B , Charbonnier B, Aoude A, Gilardino M, Harvey E, Makhoul N, Barralet J. Skeletal regeneration for segmental bone loss: vascularised grafts, analogues and surrogates. Acta Biomater 2021;136:37–55. 34626818 10.1016/j.actbio.2021.09.053

[2] Ou Z , Wei J, Lei J, Wu D, Tong B, Liang H, Zhu D, Wang H, Zhou X, Xu H, Du Z, Du Y, Tan L, Yang C, Feng X. Biodegradable Janus sonozyme with continuous reactive oxygen species regulation for treating infected critical-sized bone defects. Nat Commun 2024;15:10525. 39627239 10.1038/s41467-024-54894-8 PMC11615367

[3] Tang J , Hu J, Bai X, Wang Y, Cai J, Zhang Z, Geng B, Pan D, Shen L. Near-infrared carbon dots with antibacterial and osteogenic activities for sonodynamic therapy of infected bone defects. Small 2024;20:e2404900. 39295501 10.1002/smll.202404900

[4] Zhu Y , Yu X, Liu H, Li J, Gholipourmalekabadi M, Lin K, Yuan C, Wang P. Strategies of functionalized GelMA-based bioinks for bone regeneration: recent advances and future perspectives. Bioact Mater 2024;38:346–73. 38764449 10.1016/j.bioactmat.2024.04.032 PMC11101688

[5] Raftery RM , Castano IM, Chen G, Cavanagh B, Quinn B, Curtin CM, Cryan SA, O'Brien FJ. Translating the role of osteogenic–angiogenic coupling in bone formation: highly efficient chitosan-pDNA activated scaffolds can accelerate bone regeneration in critical-sized bone defects. Biomaterials 2017;149:116–27. 29024837 10.1016/j.biomaterials.2017.09.036

[6] Tournier P , Guicheux J, Pare A, Maltezeanu A, Blondy T, Veziers J, Vignes C, Andre M, Lesoeur J, Barbeito A, Bardonnet R, Blanquart C, Corre P, Geoffroy V, Weiss P, Gaudin A. A partially demineralized allogeneic bone graft: in vitro osteogenic potential and preclinical evaluation in two different intramembranous bone healing models. Sci Rep 2021;11:4907. 33649345 10.1038/s41598-021-84039-6 PMC7921404

[7] Pei X , Wang L, Wu L, Lei H, Feng P, Fan C, Zhou Z, Wang L, Liu M, Zhou C, Kong Q, Fan Y. Heterogeneous porosity design triggered stress reorganization to avoid intervertebral cage subsidence and promote spinal fusion. Compos Struct 2023;323:117516.

[8] Zhou J , Wang H, Virtanen S, Witek L, Dong H, Thanassi D, Shen J, Yang YP, Yu C, Zhu D. Hybrid zinc oxide nanocoating on titanium implants: controlled drug release for enhanced antibacterial and osteogenic performance in infectious conditions. Acta Biomater 2024;189:589–604. 39343288 10.1016/j.actbio.2024.09.039

[9] Li W , Su Z, Hu Y, Meng L, Zhu F, Xie B, Zhou Z, Cui S, Wang M, Wu Q, Yao S. Functional and structural construction of photothermal-responsive PEEK composite implants to promote bone regeneration and bone-implant integration. Compos Sci Technol 2024;258:110885.

[10] Sun J , Li J, Shan A, Wang L, Ye J, Li S, Zhou W. A novel multifunctional PEEK internal fixation plate regulated by gentamicin/chitosan coating. Colloids Surf B Biointerfaces 2025;245:114316. 39405951 10.1016/j.colsurfb.2024.114316

[11] Wu Y , Liu P, Feng C, Cao Q, Xu X, Liu Y, Li X, Zhu X, Zhang X. 3D printing calcium phosphate ceramics with high osteoinductivity through pore architecture optimization. Acta Biomater 2024;185:111–25. 39002921 10.1016/j.actbio.2024.07.008

[12] Qiu B , Zhao C, Pan J, Zhou Q, Yao W. Enhancing osteointegration and antibacterial properties of PEEK implants via AMP/HA dual-layer coatings. Surf Interfac 2024;51:104761.

[13] Mi L , Li F, Xu D, Liu J, Li J, Zhong L, Liu Y, Bai N. Performance of 3D printed porous polyetheretherketone composite scaffolds combined with nano-hydroxyapatite/carbon fiber in bone tissue engineering: a biological evaluation. Front Bioeng Biotechnol 2024;12:1343294. 38333080 10.3389/fbioe.2024.1343294 PMC10850574

[14] Li Q , Gao Q, Wang L, Liu L, Yang H, Song Y. Comparison of long-term follow-up of n-HA PA66 cage and PEEK cage of lumbar interbody fusion in multi-level degenerative lumbar diseases: a stepwise propensity score matching analysis. Orthop Surg 2024;16:17–28. 37953456 10.1111/os.13929 PMC10782257

[15] Qiao B , Zhou D, Dai Z, Zhao W, Yang Q, Xu Y, Li X, Wu J, Guo S, Jiang D. Bone plate composed of a ternary nanohydroxyapatite/polyamide 66/glass fiber composite: biocompatibility in vivo and internal fixation for canine femur fractures. Adv Funct Mater 2019;29:1808738.

[16] Zou Q , Li J, Niu L, Zuo Y, Li J, Li Y. Modified n-HA/PA66 scaffolds with chitosan coating for bone tissue engineering: cell stimulation and drug release. J Biomater Sci-Polym Ed 2017;28:1271–85. 28402219 10.1080/09205063.2017.1318029

[17] Wang W , Zhang B, Li M, Li J, Zhang C, Han Y, Wang L, Wang K, Zhou C, Liu L, Fan Y, Zhang X. 3D printing of PLA/n-HA composite scaffolds with customized mechanical properties and biological functions for bone tissue engineering. Compos B Eng 2021;224:109192.

[18] Li J , Wei J, Li A, Liu H, Sun J, Qiao H. A dual peptide sustained-release system based on nanohydroxyapatite/polyamide 66 scaffold for synergistic-enhancing diabetic rats' fracture healing in osteogenesis and angiogenesis. Front Bioeng Biotechnol 2021;9:657699. 34124019 10.3389/fbioe.2021.657699 PMC8188490

[19] Song P , Li M, Zhang B, Han GX, Wang Y, Zhou L, Guo W, Zhang L, Li Z, Zhou Z, Fan C, Zhang YX. DLP fabricating of precision GelMA/HAp porous composite scaffold for bone tissue engineering application. Compos B Eng 2022;244:110163.

[20] Kundu K , Afshar A, Katti DR, Edirisinghe M, Katti KS. Composite nanoclay-hydroxyapatite-polymer fiber scaffolds for bone tissue engineering manufactured using pressurized gyration. Compos Sci Technol 2021;202:108598.

[21] Huang J , Wei J, Jin S, Zou Q, Li J, Zuo Y, Li Y. The ultralong-term comparison of osteogenic behavior of three scaffolds with different matrices and degradability between one and two years. J Mater Chem B 2020;8:9524–32. 32996978 10.1039/d0tb01987a

[22] Li X , Zou Q, Wei J, Li W. The degradation regulation of 3D printed scaffolds for promotion of osteogenesis and in vivo tracking. Compos B Eng 2021;222:109084.

[23] Cai B , Jiang N, Zhang L, Huang J, Wang D, Li Y. Nano-hydroxyapatite/polyamide66 composite scaffold conducting osteogenesis to repair mandible defect. J Bioact Compat Polym 2019;34:72–82.

[24] Liu Z , Zhang M, Wang Z, Wang Y, Dong W, Ma W, Zhao S, Sun D. 3D-printed porous PEEK scaffold combined with CSMA/POSS bioactive surface: a strategy for enhancing osseointegration of PEEK implants. Compos B Eng 2022;230:109512.

[25] Jung H-D , Jang T-S, Lee JE, Park SJ, Son Y, Park S-H. Enhanced bioactivity of titanium-coated polyetheretherketone implants created by a high-temperature 3D printing process. Biofabrication 2019;11:045014. 31365916 10.1088/1758-5090/ab376b

[26] Yang X , Gao J, Yang S, Wu Y, Liu H, Su D, Li D. Pore size-mediated macrophage M1 to M2 transition affects osseointegration of 3D-printed PEEK scaffolds. Int J Bioprinting 2023;9:755. 10.18063/ijb.755 PMC10339443 37457949

[27] Zhou Y , Gao X, Zhao M, Li L, Liu M. Three-dimensional printed sodium alginate clay nanotube composite scaffold for bone regeneration. Compos Sci Technol 2024;250:110537.

[28] Hu J , Wei J, Liu J, Yuan L, Li Y, Luo X, Li Y, Li J. A novel strategy for fabrication of polyamide 66/nanohydroxyapatite composite bone repair scaffolds by low-temperature three-dimensional printing. ACS Biomater Sci Eng 2024;10:4073–84. 38752228 10.1021/acsbiomaterials.4c00457

[29] Zeng Z , Song P, Gui X, Zhang B, Zhao L, Feng P, Deng Z, Wang L, Wei W, Fan C, Wu Y, Kong Q, Fan Y, Zhou C, Song Y. 3D printed nanohydroxyapatite/polyamide 66 scaffolds with balanced mechanical property and osteogenic ability for bone repair. Mater Des 2024;241:112896.

[30] Wang G , Yuan N, Li N, Wei Q, Qian Y, Zhang J, Qin M, Wang Y, Dong S. Vascular endothelial growth factor mimetic peptide and parathyroid hormone (1–34) delivered *via* a blue-light-curable hydrogel synergistically accelerate bone regeneration. ACS Appl Mater Interfaces 2022;14:35319–32. 35881151 10.1021/acsami.2c06159

[31] Reid IR , Billington EO. Drug therapy for osteoporosis in older adults. Lancet 2022;399:1080–92. 35279261 10.1016/S0140-6736(21)02646-5

[32] Ota M , Takahata M, Shimizu T, Momma D, Hamano H, Hiratsuka S, Amizuka N, Hasegawa T, Iwasaki N. Optimal administration frequency and dose of teriparatide for acceleration of biomechanical healing of long-bone fracture in a mouse model. J Bone Miner Metab 2019;37:256–63. 29721806 10.1007/s00774-018-0930-3

[33] Chen T , Wang Y, Hao Z, Hu Y, Li J. Parathyroid hormone and its related peptides in bone metabolism. Biochem Pharmacol 2021;192:114669. 34224692 10.1016/j.bcp.2021.114669

[34] Song P , Gui X, Wu L, Su X, Zhou W, Luo Z, Zhang B, Feng P, Wei W, Fan C, Wu Y, Zeng W, Zhou C, Fan Y, Zhou Z. DLP fabrication of multiple hierarchical biomimetic GelMA/SilMA/HAp scaffolds for enhancing bone regeneration. Biomacromolecules 2024;25:1871–86. 38324764 10.1021/acs.biomac.3c01318

[35] Li G , Liu S, Chen Y, Zhao J, Xu H, Weng J, Yu F, Xiong A, Udduttula A, Wang D, Liu P, Chen Y, Zeng H. An injectable liposome-anchored teriparatide incorporated gallic acid-grafted gelatin hydrogel for osteoarthritis treatment. Nat Commun 2023;14:3159. 37258510 10.1038/s41467-023-38597-0 PMC10232438

[36] Janmohammadi M , Doostmohammadi N, Bahraminasab M, Nourbakhsh MS, Arab S, Asgharzade S, Ghanbari A, Satari A. Evaluation of new bone formation in critical-sized rat calvarial defect using 3D printed polycaprolactone/tragacanth gum-bioactive glass composite scaffolds. Int J Biol Macromol 2024;270:132361. 38750857 10.1016/j.ijbiomac.2024.132361

[37] Janmohammadi M , Nourbakhsh MS, Bahraminasab M, Tayebi L. Enhancing bone tissue engineering with 3D-Printed polycaprolactone scaffolds integrated with tragacanth gum/bioactive glass. Mater Today Bio 2023;23:100872. 10.1016/j.mtbio.2023.100872 PMC10709082 38075257

[38] Bahraminasab M , Doostmohammadi N, Talebi A, Arab S, Alizadeh A, Ghanbari A, Salati A. 3D printed polylactic acid/gelatin-nano-hydroxyapatite/platelet-rich plasma scaffold for critical-sized skull defect regeneration. Biomed Eng Online 2022;21:86. 36503442 10.1186/s12938-022-01056-w PMC9743557

[39] Zeng Z , Wang L, Qu B, Gui X, Zhang B, Deng Z, Qin Y, Li Z, Li Q, Wang L, Fan Y, Zhou C, Song Y. Enhanced osteogenesis and inflammation suppression in 3D printed n-HA/PA66 composite scaffolds with PTH(1-34)-loaded nPDA coatings. Compos B Eng 2024;282:111566.

[40] Sun H , Zhang C, Zhang B, Song P, Xu X, Gui X, Chen X, Lu G, Li X, Liang J, Sun J, Jiang Q, Zhou C, Fan Y, Zhou X, Zhang X. 3D printed calcium phosphate scaffolds with controlled release of osteogenic drugs for bone regeneration. Chem Eng J 2022;427:130961.

[41] Song P , Hu C, Pei X, Sun J, Sun H, Wu L, Jiang Q, Fan H, Yang B, Zhou C, Fan Y, Zhang X. Dual modulation of crystallinity and macro-/microstructures of 3D printed porous titanium implants to enhance stability and osseointegration. J Mater Chem B 2019;7:2865–77. 32255089 10.1039/c9tb00093c

[42] Kapat K , Srivas PK, Dhara S. Coagulant assisted foaming a method for cellular Ti6A14V: influence of microstructure on mechanical properties. Mater Sci Eng A Struct Mater Prop Microstruct Process 2017;689:63–71.

